# Associations between Pet Care Responsibility, Companion Animal Interactions, and Family Relationships during COVID-19

**DOI:** 10.3390/ani12233274

**Published:** 2022-11-24

**Authors:** Linda Charmaraman, Elizabeth Kiel, Amanda M. Richer, Alyssa Gramajo, Megan K. Mueller

**Affiliations:** 1 Wellesley Centers for Women, Wellesley College, Wellesley, MA 02481, USA; 2 Cummings School of Veterinary Medicine, Tufts University, North Grafton, MA 01536, USA

**Keywords:** child-pet interactions, adolescent-pet interactions, family relationship quality, pet ownership identity, parent–child relations, pet caretaking, pet companionship, mixed methods

## Abstract

**Simple Summary:**

For families with children during the COVID-19 pandemic, it is crucial to explore how both youth and parents view their roles with regard to the shared caretaking of pets. We present findings from a U.S. based study of adolescents and parents regarding pet care responsibility. As part of a broader longitudinal study, we analyzed survey data from 567 pet-owning adolescents and a subset of 356 dog owning adolescents aged 10–17. We also conducted 31 in-depth interviews with parents of adolescents from the same study. Adolescents who reported more pet caretaking responsibilities were more likely to spend time with pets to cope with stress and to have improved family relationships during the COVID-19 pandemic. For dog owners only, increased levels of responsibility for the pet was significantly associated with a higher likelihood of identifying as a pet owner. Qualitative findings showcase the range of parental expectations and adolescent initiative around pet caretaking. Our study highlights the continued importance of pet companionship during the adolescent years as they develop their identities as responsible pet owners.

**Abstract:**

For families with children during the COVID-19 pandemic, it is crucial to explore how both youth and parents view their roles with regard to the shared caretaking of pets. While most human–animal interaction studies examine adult or early childhood samples, our focus was on adolescent development. We present findings from a U.S. based mixed-method study of adolescent surveys and parent interviews regarding pet care responsibility. As part of an ongoing longitudinal study, we analyzed survey data from 567 pet-owning adolescents and a subset of 356 dog owning adolescents aged 10–17 as well as 31 in-depth interviews with parents of adolescents from the same study. Higher reported pet caretaking responsibilities was significantly associated with a preference for spending time with pets when stressed and improved family relationships during the COVID-19 pandemic for both pet owners and dog owners. For dog owners only, increased levels of responsibility for the pet was significantly associated with a higher likelihood of identifying as a pet owner. Qualitative findings showcase the range of parental expectations and adolescent initiative around pet caretaking. Our study highlights the continued importance of pet companionship during the adolescent years as they develop their identities as responsible pet owners.

## 1. Introduction

Approximately 70% of households in the US own a pet [1]. In the first months of COVID-19 pandemic and the resulting stay-at-home orders and social distancing mandates, public interest in pet adoption spiked significantly around the world [2,3]. According to the ASPCA, nearly 20% of households (approximately 23 million) in the US acquired a dog or cat between March 2020 and May 2021 [4]. Households with children under 18 years old were 1.83 times more likely to have acquired a pet than those without [5].

Given the exponential increase in “pandemic pets”, the media reported on rising concerns related to post-pandemic pet abandonment [6,7], concerns about pets returning in large numbers to shelters after the early months of the COVID-19 pandemic stemmed from a variety of factors including a return to in-person work and families having less time spent with pets, pandemic-related economic hardship, and a pets preventing travel after bans are lifted [4,8]. Differences in pet relinquishment also varied between groups—households with children under 18 years old were more likely to relinquish an animal after the early months of the pandemic [5].

As reflected in the increased focus on the dynamics of pet ownership during the pandemic, understanding the role of pets in the family system is critical to supporting both human and animal health and well-being. In particular, for families with children, it is crucial to explore the types of interactions youth are having with their pets, and how both youth and parents view their role with regard to the care of the pet.

### 1.1. Identifying as a Pet Caretaker: Are They Family Members or Household Chores?

The term “pet” has evolved over time to signify special animals that family members take care of not only physically but in quality of life [9]. These elements of defining pets are linked to how individuals view their relationships as pet caretakers. Pets are often viewed as valued family members; one study found that 77% of adults believe that their dog or cat is a member of their family [10]. Other studies conducted in the U.K. demonstrated that pets can be significant others for children and adolescents [11], particularly those with no siblings [12] or who are youngest siblings [13]. An individual’s conception of their relationship to their pet can significantly impact the nature of the relationship. For example, one study [14] of pre-adolescents who anthropomorphize their pets (e.g., give them presents) found that pets offered reflected appraisals of their self-worth, often acting as a “best friend”. Research has found that people who are more likely to anthropomorphize their pets are more likely to perceive that these animals provide them social support, thus improving their mental and physical health [15]. Melson contends that the caretaking of pets may be an important culturally sanctioned form of caretaking for children (especially boys) given that children are typically thought of as recipients of care [16]. Indeed, the more a child understands the specific requirements and participate in taking care of a pet, the stronger the bond between them [16].

Studies are scarce that examine a child’s personal identification as a pet owner as opposed to a pet in the family that everyone owns. Fifield and Forsyth demonstrated that 63% of pet-owning families in their sample in New Zealand identified their early adolescent child as the sole owner of at least one pet [17]. Those who are more likely to have a pet of their own and be the sole “owner” of the family pet include only children, children with one sibling, children who have their own room, and children living in a household with more than one pet or multiple pet species [17]. The most common reasons that parents acquired a pet for their children were (1) the child wanted their own pet, (2) to teach responsibility and care, and (3) to provide companionship and physical comfort. Interestingly, children of parents who had acquired a pet to teach them a sense of responsibility in the end cared for pets less often than those who wanted the pet for other reasons (e.g., they desired the pet). One study in the U.K. reported that identifying one sole owner of the pet is often vague—sometimes children are explicitly told that this is their pet and their responsibility. More often than not, children assign the role of pet owner to the one in the family who asked for the pet [18].

Adolescence in particular is a fertile period when identity development takes place and identifying as a pet owner can be a core aspect of one’s social identity. Prior research revealed that early adolescents place pets almost as high as parents and friends as sources of validation and positive self-esteem [19]. However, some adolescents are socialized by adults to believe that playing or cuddling with one’s pet becomes less age-appropriate (except in the case of dog-owners). As tweens turn into teens, they may be mature enough to engage in outdoor activities with their pet dogs and peers, whereas younger adolescents may prefer to stay at home with their fish, small animals, and birds [12]. Other adolescents may confer owning a dog as giving them higher social status amongst their peers, which can solidify their desire to identify as a pet owner [20]. More research is needed to understand this identity development of pet ownership during adolescence, and whether it is associated with a sense of pet responsibility or if it depends on the type of pet.

In 2019, the U.K. Avon Longitudinal Study of Parents and Children (ALSPAC) found that, for children ages 0 to 18, ownership of all types of pets (other than cats and dogs) peaked at age 11 (80%) and decreased through adolescent years [21]. However, cat ownership among adolescents remained constant (approximately 30%) and dog ownership increased between 11 and 18 [21]. There is some evidence to suggest that children become less involved with their pets as they get older, with one cross-sectional survey study indicating that 75% of older adolescents (mean age 15.9) reporting rather little interaction with their pets [22]. Given the need for increasing autonomy and peer interaction in the adolescent years, it is not surprising that the relationship with companion animals can shift over time [23]. There are a few theories that attempt to explain why children begin to lose interest or sense of attachment to their pets as they grow older into the adolescent stages. One explanation is that as one gets older into adulthood, there is a tendency for people to impart the need to avoid looking too attached to pets for fear of appearing childish or unable to form bonds with humans [24]. Another theory is that children go through a natural process of desensitization or a form of psychological distancing as they grow apart from their pets [25]. Prior research has also shown that the expectations of young children’s ability to care for pets beyond cuddling and playing shift as they grow older and can fully understand the caretaking responsibilities, with parents less likely to limit the type and range of caretaking chores [18]. The researchers observed that younger children often avoided certain caretaking tasks deemed unpleasant or revealed being afraid of the pet (or the pet doesn’t like them), noting that the age of the child could be a consideration of parent’s expectations. In this study the researchers had a wide range of children aged 8 to 13, yet did not disaggregate the older children from the younger ones when drawing their conclusions. Despite this general trend of a waning interest over time, there are also studies that indicate that adolescence is a particularly crucial time for the availability of conflict-free relationships with a pet [26] and feeling a sense of autonomy and heightened self-esteem through their pet relationships [27]. More research is needed to understand how pet caretaking evolves within families from multiple perspectives during that developmental transition between the childhood and tween years and the tween and teen years.

### 1.2. Navigating Pet Caretaking Responsibilities within Families

Studies have showcased that the inclusion of a pet within a household and the responsibilities that go with it (e.g., feeding, watering, grooming, exercising, and training) strengthens the bond between child and animal, particularly when they have no siblings or if they are left home alone while parents work (e.g., Guerney, 1991 [28]). Building healthy relationships between child and pet depends a great deal on how parents and other adults role model these caretaking behaviors [29]. In fact, one study demonstrated that the more effective parents were in providing guidance on pet caretaking, the more their child was able to learn how to problem solve [30]. There is little known about the ways that adolescents conceptualize pet care responsibility. One study [31] found that, early in life, most young children (62%) do not understand the difference in the role of human care in domestic pets versus wild animals. Adolescents, conversely, are keenly aware of the role of humans in pet care, with a majority citing “learning about responsibility and animal life” as one of the most important benefits of child pet ownership [32].

Prior literature on pet caretaking within families with children is rather outdated. One seminal study in the U.K. [32] found that nearly all adolescents (ages 10 to 15) surveyed (89.4%) reported to have cared for an animal for “a long period of time” before. Another study in New Zealand demonstrated that 14% of families reported an equal workload of pet responsibilities across all family members, with 38% of children participating in caring for their favorite pet an equal or greater amount of time compared to their parents or siblings [17]. Further, mothers were found to be responsible for the majority of pet care in 34% of families, reporting that they did at least half of the work in 41% of households. In 8% of households, fathers did the majority of the pet care chores [17]. Past research has also found that the distribution of petcare roles evolve throughout developmental “stages” of the nuclear family. In families with school aged children, the responsibilities were split between the child and parent. In families with teenagers, the child held the majority of petcare responsibilities [33].

Both parent and child attitudes appear to have a significant influence on the division of pet caretaking labor in their families. One study in the U.K. found that 94% of parents find pet caretaking skills important and beneficial to their children [32]. On the other hand, children may ask their parents for pets, though they may not fulfill their responsibilities willingly or enthusiastically, creating a difficult environment for parents to create and implement petcare plans [34]. In addition, one U.K. study found that some parents limit the types of pet care responsibilities their children take on, based upon the belief that affection and playing with a pet were age-appropriate “responsibilities” for children or any perceived risks or harms that the tasks may cause the child or pet [18]. Previous research has found that girls are more likely to perceive pet caretaking as the responsibility of a pet “owner”, regardless of whether they are a parent or child [32]. Given the lack of attention to pet caretaking responsibilities in more recent years, more research is needed to understand adolescents’ role in the current social climate.

### 1.3. Evolving Pet Caretaking Needs and Family Relations during the COVID-19 Pandemic

The nature of attachment to pets and pet-related responsibilities shifted for many families at the beginning of the COVID-19 pandemic due to a seismic change in both daily routine and amount of time spent at home together. Early in lockdown, the majority (79.5%) of dog owners reported that the pandemic had changed their dog’s daily routine, including variations in the amount of time pets spent alone, decreased walking activity, and more frequent playtime [35]. However, a recent study found that dog owners were more likely to have a walking routine and spend time outdoors [36]. Further, 9 in 10 Households with both dogs and children found that they were spending more time together than they did pre-pandemic times in both the U.S. and the U.K. [35,37]. Adults who were suddenly expected to take care of their children and pets at the same time, while also mediating their interactions, were forced to balance responsibilities in new and challenging ways [9].

As pets can be considered part of the nested family system, responsible and caring interactions with companion animals can be one measure of healthy family functioning [38]. Prior studies in the U.K. and Scotland have shown that during times of family relationship disruptions, pets can be one of the few sources of reliable, consistent, and responsive affection available to children [39,40]. Pets can be a main source of comfort when lonely or distressed—for girls, pets often offer an audience for self-disclosure and for boys, pets are an activity companion and someone to spend time with [20]. At the other end of the spectrum, problem behaviors of children and pets can be exacerbated by the stresses of confinement and feeling stir crazy [9]. During the pandemic, pets were at times a distraction for families as they navigated their children’s online schooling giving pets more opportunities to become problematic with the lack of supervision [37]. This was especially relevant during the lockdown periods because many families with children depend on extended family for childcare, petcare, or both and these lifelines were severed during the height of the pandemic [37], which may have put strains on family relationships.

The social lockdowns during the COVID-19 pandemic disrupted typical family interactions and processes, requiring a renegotiation of pet-related and non-pet-related responsibilities in the home. For instance, a study [41] found that families experienced a loss of their usual community ties to support their pet caretaking, especially if it would potentially expose these friends to contracting illnesses. In terms of positive pandemic effects, parents observed pets were a predominantly positive presence in their household (65%) and 28% reported having no effect [37]. When describing the types of interactions with pets, parents observed that children were most often playing with their pet, followed by cuddling and taking care of their pet’s physical needs, and finally, keeping their children “busy”. Parents also observed that their child used them as coping and comforting mechanisms, to reduce loneliness, to be a distraction, and to reduce stress and anxiety [37]. In this study, the children of the parents ranged widely in age from 0 to 27 therefore more studies about the particularly developmental period of adolescence are needed, given that this was a vulnerable segment of the population during the social distancing of the lockdowns. Furthermore, although there is a growing number of studies that focus on pandemic effects for the child or the parent or the pet separately, there is scarce research on the importance of the family system and relationship dynamics that may have been affected during this social distancing period.

### 1.4. Current Study

To fill in gaps in the field concerning adolescent pet responsibility, in-depth qualitative and mixed-method research is needed to obtain more detailed accounts of different family members’ perspectives of adolescents’ pet relationships and caretaking, e.g., [20,42]. Given that the majority of research on youth pet caretaking responsibilities is out-of-date and rely on one informant in the family, we present findings from a U.S. based mixed-method study of adolescent surveys and parent interviews regarding pet care responsibility. With most human–animal interaction studies focused on adult or early childhood samples, we highlight an overlooked period of development, particularly when children may shift in their identity as a pet owner/caretaker. Although there are prior studies that show how pets can alleviate distress [43], there are limitations in our knowledge of how *adolescents* cope with their stress by leaning on a pet for mental health benefits and whether the physical and mental act of taking care of pets is related to these coping behaviors. Much of the work on human-pet interactions has been conducted in pre-COVID-19 pandemic times, thus studies are emerging on how the social distancing period affected families with pets of different types. The following mixed-method research questions address our study aims:

Quantitative:

1a. From the adolescents’ perspective, how is companion pet responsibility related to family relationships during the COVID-19 pandemic, such as identifying as a pet owner and coping strategies when stressed?

1b. Are there any differences between pet owning adolescents and dog owning adolescents?

Qualitative:

2a. How do parents socialize their adolescents to take care of their pets? What kinds of pet responsibilities do parents report adolescents take part in? How willingly are they participating in caretaking?

2b. How do parents characterize the bonds between adolescents and their pets? Are pets thought of as family members or another household chore by adolescents?

## 2. Materials and Methods

### 2.1. Quantitative Procedures

#### 2.1.1. Adolescent Survey Recruitment

As part of a more extensive, longitudinal study of social and digital technology use among young adolescents, in Fall 2020 we recruited middle school students (grades 6–8) and high school students (grades 9–10) located in several Northeast United State school districts. School sites were selected based on school enrollment size, internet accessibility, and diverse racial/ethnic, and socioeconomic composition. Working with school principals and liaisons, we distributed informed consent/opt-out forms to parents (available in English, Spanish, and Portuguese). Parents received this information through paper flyers, school e-newsletters, parent email contact lists, parent-teacher organizations, and direct emails. The survey was distributed using Qualtrics during a dedicated in-person advisory period or health/wellness class, lasting up to 60 min. The dates of the survey were pre-scheduled with school administrators. Due to the COVID-19 pandemic and the remote learning period, survey data collection was spread out over the course of several days to capture all student cohorts. Some students were still fully remote and therefore completed the survey at home. The Qualtrics survey link was distributed to students in the Google Classroom platform, allowing any students absent during the survey period to participate from home. Due to state-wide restrictions, research staff was unable to enter the school sites in person. As a result, we relied on school teachers and staff to administer the survey. Study staff remained available via video conferencing to answer questions and troubleshoot technical issues. School sites received an honorarium along with gift card incentives for site coordinators. Adolescents that participated in the survey were entered into a gift card raffle for multiple 25$ gift certificates.

#### 2.1.2. Survey Samples

*Pet Owner Sample.* Pet owning students (*n* = 567) were 55% female, 44% male, and 1% non-binary gender or missing, and represented diverse racially/ethnically backgrounds, with 62% identifying as White, 13% were Hispanic, 5% were American Indian, 4% were Black, 3% were Asian, 2% Middle Eastern, 6% multiracial, and 5% other/unknown race/ethnicity (Refer to Table 1). Sixty-three percent of students reported owning a dog and forty percent of students received free or reduced price lunch. Student age ranged from 10 to 17 years old with an average age of 13.09 years.

*Dog Owner Sample.* Dog owning students (*n* = 356) were 55% female, 45% male, and represented diverse racially/ethnically backgrounds, with 66% identifying as White, 10% were Hispanic, 5% were American Indian, 3% were Black, 3% Middle Eastern, 2% were Asian, 5% multiracial, and 7% other/unknown race/ethnicity (Refer to Table 1). Thirty-nine percent of students received free or reduced price lunch. Student age ranged from 10 to 17 years old with an average age of 13.24 years.

### 2.2. Quantitative Measures

#### 2.2.1. Pet Ownership Characteristics

Pet ownership was measured by a single item asking adolescents if they had a pet (Yes/No). Dog ownership was measured by a follow up question to pet ownership about the species of the pet. If the adolescent had more than one pet, they were asked to indicate the species of their favorite pet. Species response options included: “*Dog*”, “*Cat*”, “*Reptile/Fish*”, “*Horse*”, “*Other animal*”, or “*Other*”. If an adolescent reported having a “*Dog*”, they were coded as a dog owner (1). If the adolescent indicated having any other type of pet, they were coded as a non-dog owner (0).

#### 2.2.2. Adolescent-Pet Interactions and Coping with Stress

Pet caretaking responsibility was measured using a single item asking adolescents how much responsibility they have caring for their pet at home on a 6-point scale ranging from “*Little to none*” (0) to “*The most*” (5). Adolescents were asked how much they agree with the following coping strategies to manage stress: spending time with a close friend, family, or pet(s); exercising/sports; spending time outdoors or in nature; being alone; using technology, such as watching favorite movies/TV shows, playing video games, social media, etc. Spending time with pets when stressed was measured using a single item on a 4-point scale ranging from “*Mostly Disagree*” (1) to “*Mostly Agree*” (4).

#### 2.2.3. Adolescent Identity as a Pet Owner

Pet owner identity was a single item that was part of a larger set of 10 items asking adolescents to check their top 5 identities (e.g., friend, gender, athlete, student, hobbies, etc.) that are important to them in their life. If adolescents selected “*Having a pet*” as one of their top 5 identities, they received a (1). If they did not select “*Having a pet*”, they received a (0).

#### 2.2.4. Family Relationship Quality during COVID-19

Family relationships during COVID-19 was measured with a single item asking adolescents to report how the quality of their family relationships had changed since the beginning of social distancing due to COVID-19. This item had a 5-point scale ranging from “*A lot worse*” (1) to “*A lot better*” (5).

#### 2.2.5. Covariates

*Age*. We calculated age using date of birth and date of survey completion.

*Gender*. Participants were asked whether they identified as female, male, or non-binary/other.

*Free/reduced price lunch*. As a proxy for socioeconomic status, we asked whether participants were eligible for free or reduced price lunch in their schools (Y/N). Those indicating Y are considered lower socioeconomic status compared to those who are not eligible.

### 2.3. Qualitative Procedures

#### 2.3.1. Parent Interview Recruitment

Parent interview participants were recruited from a subset of parents from the larger longitudinal study [44,45] that indicated interest in doing a follow-up interview study. We included parents with a range of parental monitoring practices, spanning from low to high. 69 interested parents were contacted via direct email correspondence. We also recruited through school-wide announcements from principals and parent referrals. Our recruitment efforts resulted in 31 parent interviews. Before scheduling the interviews, study disclosures were distributed and parents provided signed consent. All interviews were conducted via Zoom.

#### 2.3.2. Parent Interview Sample

Of the 31 interviews, 28 parents identified as mothers/female guardians, 2 as fathers/male guardians, and 1 as a non-binary parent/guardian. We confirmed demographic information from the longitudinal study at the start of the interview. 21 parents identified as White (68%), 2 Hispanic (6.5%), 2 Brazilian (6.5%), 1 Black (3%), 1 multiracial/ multiethnic (3%), and 4 unknown (13%). The majority (74%) of parents were dog owners, 23% owned cats, and 3% owned guinea pigs. Parents were asked to answer interview questions focusing on their middle school child. Most parents referred to daughters (*n* = 21) and 10 referred to sons. We confirmed the gender of the child by using the child’s self-report in the longitudinal study and the parent’s determination in the interview.

#### 2.3.3. Parent Interview Protocol

The exploratory interviews covered topics including how, when, and why they acquired their pet(s), child emotional attachment to their pet, coping and stress management, caretaking responsibilities, and social media and digital technology as it relates to the household pet. Because interviews were conducted during the height of the COVID-19 pandemic, parents were asked to discuss the impact of the COVID-19 pandemic on having or acquiring a pet, with an emphasis on their child’s remote learning experience. Parents were asked to focus on their middle school-aged child if they had more than one child and their child’s favorite pet if they had multiple pets. Interviews were conducted, recorded, and transcribed using Zoom.

#### 2.3.4. Analysis Plan

*Quantitative*. Regression analyses were used to explore the relationships between responsibility for a pet and identity, stress and family relationships within pet owners and within dog owners. Logistic regression was used to model identity and linear regression was used to model stress and family relationships. Regression models were first run separately for each outcome for pet owners (*n* = 567), excluding any students from the larger sample (N = 968) who were not pet owners. Next, regression models were run separately for each outcome for dog owners (*n* = 356), excluding students from the pet owners sample (N = 567) that did not own a dog. For each model, pet responsibility was the predictor variable and identity/family relationships/family relationships was the outcome or dependent variable. Each model controlled for age, calculated using student date of birth and date of survey completion, self-reported binary gender, and whether students received free or reduced price lunch at school. Students indicating a non-binary gender (*n* = 4) were categorized as missing, due to the low prevalence that could not be included in these analyses. For each regression model, missing data was handled using listwise deletion resulting in different sample sizes for each regression model. Sample sizes ranged from 295–538 for each model.

*Qualitative*. Zoom interviews were recorded, transcribed, and verified by the research team, and imported into NVivo software (NVivo 12 Version: Plus) [46]. An initial open coding phase by research assistants was used to develop the codebook, which included broad buckets of categories directly from the interview questions and subcategories that illustrated subthemes within those buckets. Initial nodes on Nvivo were created a priori according to the main interview questions as part of the larger study. New codes and subcodes related to the current study’s research questions were then created through both deductive and inductive processes. Discussions with team members about reactions to the interview content helped us refine the primary codes, (e.g., child pet caretaking responsibilities), develop secondary codes, (e.g., within child pet caretaking, breaking it down into types of chores, such as grooming, feeding, clean up, etc.), and identify any unexpected emerging themes, particularly after reviewing the preliminary quantitative findings. Our group process of reflexive thematic analysis [47] confirmed that the themes and subthemes were categorized and defined accurately. In the final stages, the relationship between aims, research questions, and themes helped determine the theme and subtheme salience [47], for which we calculated theme frequencies to provide transparency. We used pseudonyms for all names of parents, adolescents, and pets to protect their privacy.

## 3. Results

### 3.1. Quantitative Findings

We found no significant differences between average pet caretaking responsibility between pet owners (M = 3.25; SD = 1.24) and dog owners (M = 3.25; SD = 1.18).

#### 3.1.1. Adolescents Identifying as a Pet Owner

**Figure 1:**
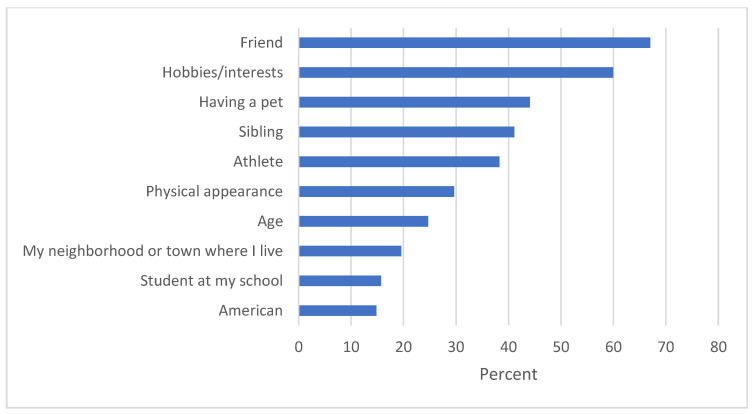
Most important social identities (or ways of defining themselves) selected by pet owners.

Within pet owners, 44% of the sample ranked being a pet owner as one of their top 5 identities, which was the third highest reported identity after identity as a friend (67%) or hobbies/interests (60%) (See Figure 1). Similarly, dog owner identity ranked as the third highest identity selected by pet owners, with 46% of dog owners selecting this as one of their top 5 identities (after identity as a friend (68%) or hobbies/interests (59%). Interestingly, identity as a sibling was lower than identifying as having a pet for both pet owners and dog owners. Identity as a pet owner was not associated with levels of pet caretaking responsibility. For dog owners, increased levels of responsibility for the pet was significantly associated with a higher likelihood of identifying as a pet owner (Odds Ratio = 1.21, *p* = 0.04).

#### 3.1.2. Coping Strategies when Stressed

**Figure 2:**
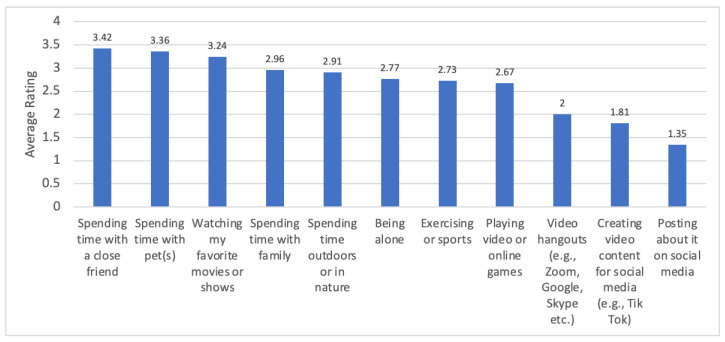
How spending time with pets compares to other coping strategies when adolescent pet owners are stressed.

Spending time with pets was the second most frequently used coping strategy when stressed for pet owners (M = 3.36; SD = 0.92) following spending time with a close friend (M = 3.42; SD = 0.87). In contrast, spending time with pets was the most frequently used stress coping strategy for dog owners (M = 3.44; SD = 0.82), with spending time with a close friend (M = 3.43; SD = 0.86) as a very close second. Other types of coping strategies such as watching favorite movies/shows, spending time with family, and being outdoors/in nature were somewhat less common than spending time with pet(s) (Refer to Figure 2). Regression results showed that higher reported pet caretaking responsibility was associated with higher ratings of spending time with pets as a stress coping strategy for both pet owners (β = 0.14, *p* = 0.004) and dog owners (β = 0.12, *p* = 0.04). Refer to Figure 2.

#### 3.1.3. Family Relationships during Pandemic

Twenty-eight percent of pet owners reported that their family relationships improved since the beginning of social distancing due to COVID-19, half of students (51%) reported the relationship stayed the same, and 21% reported a decline. Thirty percent of dog owners reported that their family relationships improved since the beginning of social distancing due to COVID-19, half of students (51%) reported the relationship stayed the same and 19% reported a decline. Higher reported pet caretaking responsibilities was significantly associated with improved family relationships during the COVID-19 pandemic for both pet owners (β = 0.11, *p* = 0.01) and dog owners (β = 0.13, *p* = 0.02). (Refer to Table 2).

### 3.2. Qualitative Findings

In this section, we describe the results of our parent interviews themes and subthemes, as detailed in Table 3.

#### 3.2.1. How Parents Socialize Adolescents to Take Care of Pets

*Conversations before getting pets.* Prior to getting a pet, some parents communicated specific expectations about pet care responsibilities to their adolescents (10 of 31). Before Diana’s eighth-grade son, Andrew, was given a dog for his birthday, she “tried to talk about” the reality of pet care responsibilities with him, as she believed that “a kid always wants a dog and they don’t realize what that means”. In addition to having this conversation with her son, Diana “gave him poop bags” as a present and hoped to enroll him in a dog training class with her to teach the dog obedience and skills. Prior to bringing their new dog home, Nancy explained to her four daughters, ages 16, 14, 12, and 10, the distinction between caring for a toy and a living creature:

*It took a long time for the dog to come into our lives, and so I think a lot of that was talked about … the responsibility… it’s a living thing that you have to take care of. This is not a bicycle you can just throw on the side of the garage or leave outside in the rain like it’s a, it’s a being, it’s a thing, and you have to take care of it… She’s a nightmare at night, running around like a psycho, chewing on things and eating my carpet and chewing on the table, and you know, we’ve had those discussions… the girls are good to clean it up before I find it because they know.*

Similarly, prior to getting a dog, Robin aimed to instill a sense of responsibility in her eighth-grade daughter, Audrey, while also giving her a sense of autonomy and influence over the decision:

*We’ve had pretty good conversations with the kids before we even considered applying for rescues, and we wanted to make sure this is a family decision and we’re not going to just take sole responsibility, you guys need to help out. So we wanted to make sure that this wasn’t going to be a honeymoon period like “oh we love the dog” and then they forget about the dog and they don’t want to take care of the dog. So we made it really clear that they’re going to have to do some work as well.*

Some parents who outlined petcare expectations for newly acquired pets did not actually expect their children to follow through on their promises. Lisa assumed that her children, including her seventh-grade son, Noah, would not take care of the dog as promised, and that pet caretaking would become “a parental responsibility, ultimately”.

*No petcare expectations.* Conversely, other parents did not outline specific petcare expectations to their adolescents before introducing them to each other. Marco’s adolescent, Lucas, is in the foster care system and, although Marco considered that “dogs teach responsibility” before Lucas joined the family, he “had no conscious plan for [him]... especially in his situation”. On the other hand, Terry felt it was unrealistic to introduce responsibilities to her children including her sixth-grade daughter, Amanda:

*Okay, [prior to getting a dog,] I envisioned me doing everything because I’m not gonna pretend like my eleven and an eight, nine year old are going to do anything… I knew it was going to be really big dog, so I knew walking the dog was out, like they were not going to be able to, he’s too big… If I say feed the dog, it takes two seconds to feed the dog, so, they’ll do that. They’ll pick up his toys… I think they’ve actually probably done more than I thought they were going to do.*

Overall, parents differed in their explicit expectations of their childrens’ petcare responsibilities prior to getting a pet. These differences appeared to be due, at least in part, to parents’ own attitudes and abilities to perform petcare, as well as their specific adolescent, family, and pet’s needs.

*Lessons surrounding petcare*. Many parents of adolescents view pet caretaking as an opportunity for their children to learn responsibility and empathy for others. Catherine encouraged her tenth-grade son, Eddie, to try to understand and empathize with, Lenny, their cat’s, needs to understand his own role and responsibilities as a caretaker:

*You know, when Eddie got older, it was easier to talk about responsibilities and, when you ask him to do something… It’s so important to [say], “I asked you to feed Lenny. Like, I’m trusting that you’re going to go downstairs and feed him, because then, if you don’t, then he has no food.*

Similarly, Cecilia believes her children developed empathy “may a little bit earlier than if [they] didn’t have a dog. When her children first got the dog, her children learned that they “[had] to care for this tiny thing and… that’s something different, besides family, a person that you know something’s wrong and they feel bad”. Understanding the needs of pets may, evidently, teach children the importance of pet caretaking and the importance of their role in these responsibilities. At other times, parents may let their adolescents learn the importance of responsibility by letting them experience negative consequences of irresponsibility. When Nancy’s sixth and eighth grade daughters, Alexa and Hannah, refused to walk their puppy in the cold weather, they learned an important lesson about responsibility when the dog reacted negatively:

*It’s really important for [the dog] to go outside so that she doesn’t have these bad behaviors because you know, in puppy years. She’s still a very young dog and, and a young child requires attention and entertainment and exercise and all those things… When [the children] shirk that responsibility, seeing the consequences firsthand what happens when the 25 min walk is a lot better than them screaming for 25 min because the dog’s going crazy and has ruined another rug.*

*COVID-19 and adolescent petcare expectations*. In some families, the COVID-19 pandemic shifted expectations of petcare responsibilities for adolescents. Cecilia’s children, including her 13-year-old daughter, Natasha, were expected to take on additional petcare responsibilities while at home during remote learning:

*Yeah I mean we always said that they had to help, that the responsibility wasn’t just going to be solely my husband and mine. And then you know with them in school, obviously the responsibility falls more on the people that are home… They never really had that set schedule, they just kind of fell into that but now… the responsibility has fallen on them because they’re home with [the dog] during the day, more than we are.*

Additionally, many families acquired “pandemic pets” during the lockdown period early in the first months of the pandemic due to family members, including adolescents, spending unprecedented amounts of time at home. Stacy was initially inspired to get a dog to be a companion for her seventh-grade daughter, Marissa, during the pandemic. Given that Marissa was taking remote classes, Stacy anticipated that it would be a “great responsibility for her to take a walk after school with a dog”. Terry’s sixth-grade daughter, Amanda, also helped take care of their family’s dog acquired during the pandemic:

*Yeah, no, I think it’s so much better [raising a dog in a pandemic] because he doesn’t have to be in a crate all day. So, like two days a week he’s in the crate in the morning, and then the dog walker comes, and then he goes back in, and then my daughter comes home and lets him out and then that’s it, you know.*

The COVID-19 pandemic, in some instances, shifted the typical delegation of petcare responsibilities in families with adolescents.

#### 3.2.2. Nature of Adolescent-Pet Bonds

*Pets as family members.* Throughout the caretaking and bonding process, we found that adolescents often feel deeply connected to their pets, with several considering them to be members of the family (9 of 31). Prior to Lucia getting her seventh-grade daughter, Jada, a dog, Lucia taught her that “a dog is like a little baby” when explaining the importance of caretaking. In effect, Lucia feels that the dog is “really like [Jada’s] little daughter”, because she “cleans her butt’... changes the water, [and] puts her food”. Likewise, Nancy’s children, including her eighth-grade daughter, Hannah, felt their dog became “an instant family member” as their bond strengthened rather quickly. In other instances, pets seem to reciprocate a caretaking relationship with adolescents. Catherine observed that her tenth-grade son, Eddie, and his cat seem to treat one another as their “baby”:

*[The cat] cleans [my son] everyday like he cuffs his head and cleans his face and his and it lifts his hair into place… [My son] can hold him like a baby and he just lays on him—he doesn’t do that to any of us… I will never forget the first time, Eddie slept at my mom’s and the cat was miserable. And he came home the next day and laid on the ground and the cat like sniffed him like… People come in and they’re like, “What’s happening?” and I’m like, “Oh, the cats cleaning Eddie…” The cat thinks that Eddie is like his baby.*

In some families, adolescents overtly refer to pets as members of the family. Robin’s tenth-grade son “refers to [the cat] as a sibling versus as just a pet”. Further, some families consider pets to be indispensable parts of the family structure. After Anne’s children, including twin sixth-grade daughters, Catie and Caroline, got a dog, she felt that their family unit was complete:

*Nothing felt complete when we didn’t have a pet with the kids so I kind of thought of the dog in terms of kind of completing our family unit and he really has fulfilled that role. And often we kind of say, “what would we do if he wasn’t here?” Like, “how would we entertain ourselves?” That kind of thing.*

*Challenges of pets in families.* Though several parents report feeling like pets have become family members, not every pet acquired fits perfectly or naturally into the family structure. While close, family-like attachments between pets and adolescents seem to confer advantages, they also may present unique challenges. Anastasia’s eighth-grade daughter, Alison, bonded “instantly when she saw” the dog but, when it started showing signs of aggression and they had to relinquish it, the situation was “hard for [the] whole family”, especially Alison. In contrast, few families distinguish between pets and family members. Although Valerie’s family considers their three guinea pigs to create a “bit of family identity”, her eighth grade daughter, Julia, sees them as pets rather than friends.

#### 3.2.3. Fulfillment of Pet Caretaking in Families with Adolescents

*Adolescents volunteering to take care of pets.* We found that adolescents perform a variety of pet caretaking responsibilities with a range of willingness, based on factors including their attitude towards the pet, their parents’ orders and expectations, and the established family dynamic. In some cases (15 of 31), adolescents are enthusiastically willing to help care for their pet. Janet’s thirteen-year-old son, Brian, takes initiative early in the morning:

*Sometimes, even before he wakes up before me. Like this morning, for instance, he woke up before I did, and he had already changed both the [cat’s] water and food. And he knows the exact measurements of food that he gives her from him watching me and my oldest daughter doing it, so he knows exactly the amount of scoop to put in her bowl…This morning was the second, maybe third, time he’s done that, without me being there watching.*

Anastatia’s eighth grade daughter, Alison, would “feed [the dog] whenever we needed to”, volunteering all of her free time to “do anything that would be needed in taking care of [the dog]”. Marcella’s eighth-grade son, Carson, enjoys the job of taking care of a pet and does not see it as an obligation:

*When he was little he loved to feed her and take her for a walk and even now, that’s like, I don’t even have to ask him… But I mean it’s one of those things that he doesn’t really think is a chore because it’s just a thing that he does every day like, “Oh, I have to take care of the animals”. It’s never something that I asked him to do.*

Some parents set firm initial expectations for pet caretaking responsibility for their adolescents which they follow closely and enthusiastically. Nancy’s family “run a tight ship” in terms of petcare responsibilities, though her children are “very good” about taking care of the dog, even asking their mother to “[try] to get another dog”.

*Blend of volunteering and assignment to take care of pets.* While some adolescents demonstrated a personal, internal motivation to care for their pets, several adolescents completed a blend of voluntary tasks and assigned explicit assignments. Marco reports that, although his thirteen-year-old son, Lucas, has been assigned to feed the dogs breakfast and dinner, there are other petcare responsibilities that “he’s come to on his own. Unless it’s picking up poop or vomit, which he will absolutely not take part in even though he’s been asked”. Although Marcella’s eighth-grade son, Carson, volunteers to take care of the dog, he is solely responsible for the pet turtles:

*His turtles are his sole responsibility. I don’t take care of them at all. He feeds them, he cleans their tank, he plays with them. So, that’s his completely. The rest of the animals, he takes care of them at least 50% of the time. When my daughter’s home, they actually fight about who’s going to feed the dog or who’s going to walk the dog, which I think is fine.*

On the other hand, some families do not have explicit rules and, instead, follow an ad hoc approach to fulfilling pets’ needs. Marina’s children, including her eighth grade daughter, Mila, do not follow a regimented schedule to take care of their dog, Theo. Instead, Marina assigns Mila and the others tasks when they arise, while Mila also takes initiative to complete pet care tasks voluntarily:

*So if you know, like if, if I am tired and I’m like, “Mila, take Theo out”, you know stuff like that. And they’re fine with that, so yeah I don’t think there are rules. And, ok, Mila is kind of like on top of things in general. Like, she’s responsible and mature and so… Mila makes sure that he gets his, for example, his medicine. Like he has something monthly.*

*Adolescents assigned to take care of pets.* For other adolescents, pet caretaking is simply an obligation. Ellis’s children, including his fourteen-year-old daughter, Madeline, are assigned a chore chart to keep track of the children’s daily cat care responsibilities:

*Both of our girls, part of their chores are changing the cats’ water, changing their litter, feeding them every day, so they have a decent amount of their caretaking… I actually divide it for them. I made up a chore chart. So they have to do the cat litter together. My older child takes care of the changing of the water, and then they each do one feeding per day.*

Ana’s children, including her twelve-year-old daughter, Gabby, also must complete tasks on a chore chart to care for their two cats and four birds. Ana reports that she and her partner devised a system where “the chores change every month, so everybody will see, you know, the kitty litter, the food, the water”. As the oldest child, Gabby is also responsible for bathing and grooming the cat. Other adolescents are, simply, not fond of pet caretaking tasks that they find uninteresting or unsavory. Michelle’s seventh-grade daughter, Marissa, helps her clean the guinea pig cages every two weeks, though she does so “reluctantly”, seeing it is an unpleasant chore.

*Parent petcare responsibilities in families with adolescents.* In few families with adolescents, petcare responsibilities fall primarily to the parents. Rachel, who has two children, including her seventh-grade, Jenna, is the primary caretaker of the dog with occasional assistance from her husband:

*Yeah, I mean if I’m not around my husband, happily, you know we’ll do the litter, feed her, but if I’m around she usually follows me. She has to take thyroid medication, so you know we drop a little pill, and all that stuff so, yeah, usually I’m the primary.*

Some parents who primarily care for the family pet wish their adolescents would contribute more to the caretaking. Trisha wishes that her children, ages 15, 13, and 12, would take more responsibility caring for their dog, Peanut:

*I would like them to even take more initiations of like, I hate to say it, but like to clean up after the pets. I mean you can’t just do it twice a year. So I think that would be the only thing [to improve upon]— for them to take more responsibility.*

*Parent expectations of adolescent petcare.* Several parents reported expecting to care for the pet prior to getting it, suspecting that their children would not be committed to their assigned or promised responsibilities. Cecilia was told at the shelter that “You know your kids are going to like this dog for five minutes and the responsibility is going to be yours”. Instead, she has been pleasantly surprised that the children have developed a strong bond with the dog, reporting that, “She’s the first thing that they say hi to when they come home, when they wake up, and the last thing that they say goodnight to them and they go to bed”.

Other parents expected their children to put more effort towards petcare than they, in reality, put forth. Kim is a disappointed that her sixth grade son, Stephen, does not care to put more effort into providing for their dog’s emotional needs:

*And it’s not that they don’t love her they do, but they’re not devoted to her care, you know? It’s more of an, “oh she’s here and let’s pet her”… In terms of meeting her emotional needs, I don’t see that... She loves to have someone throw the ball, take her outside and throw the ball with her and they never do it.*

Similarly, Marina was surprised that her children, including her eighth-grade daughter, Mila, did not walk the dog as much as they had promised prior to acquiring one. Marina reports that she is “kind of new” to pet ownership with adolescents and did not anticipate that she would have to fulfill some of those responsibilities herself.

*Pet relationships and coping strategies.* For some adolescents, pet caretaking and, subsequently, close pet relationships, have a positive impact on their lives. For Diana’s eighth-grade son, Andrew, the family’s two cats and one dog were important for providing entertainment and companionship during lockdown, effectively “boosting morale during the pandemic”. Similarly, Cecilia felt that the sense of responsibility her seventh-grade daughter, Natasha, felt towards their do, Sydney, led to a close, emotionally supportive adolescent-pet relationship:

*I think that having a pet has taught her responsibility. I think that you know emotionally like you know your pet… they sense when things are not okay. And she does. I don’t know that all pets are as intuitive as is she is, but she does. Like so she’ll sit with you, like last time we were going down the street and Abby came home early and I came in and I was like, “What are you doing?” And she was like, “I was just cuddling with Sydney”. It’s just a relationship that is so great for her it’s so great, for all of them.*

*Pet versus dog-owning adolescents*. For many dog-owning adolescents, their pet or pets are more of a source of emotional comfort as compared to other types of pets. For instance, Ben’s thirteen-year-old daughter, Stephanie, for instance, has a hard time making friends and is “a pretty guarded person” though she is “very affectionate” with their dog, Coco. Ben hopes, for his children, that the “comfort and feeling responsible, you know a responsible pet owner… can translate that confidence into the rest of their lives”. Jean reports that her twin sixth grade daughters, Angela and Samantha, began to “talk to [the dog] like she’s a human” during the pandemic lockdown. On the other hand, Valerie’s eighth grade daughter, Julia, does not feel emotionally supported by their guinea pigs. Julia feels if she, instead, had a dog, that she would “take to the dog and tell the dog [her] feelings” though “she wouldn’t do that with the guinea pig or the hamster”.

## 4. Discussion

Our mixed-methods findings highlight the unique importance of understanding the adolescent pet owning years from the perspectives of adolescents and their parents, particularly in the under-researched area of responsibility around pet caretaking, and how these aspects of the youth-pet relationship can intersect with family dynamics. Prior research [12,13,14,15,16] has already established that younger children often feel so bonded to their pets that they are considered significant others, members of the family, and best friends, however little was known about what adolescents feel about their identity as pet owners and the role they play in taking care of their animal companions. Our survey data showcased that being a pet owner was among the top 3 most important social identities for adolescents aged 10–17—even more important than being a sibling. Pet ownership identity did not predict degree of engagement in pet caretaking, however, dog owners who had higher pet caretaking responsibilities were more likely to identify with being a pet owner. This finding corroborates past work, e.g., [20] suggesting that dog owners may identify with their dogs on a different level compared to other types of animal pets. It may be that actively participating in the care of a pet supports increased connection with the pet and incorporating their role as caretaker into their identity. Our qualitative results aligned with the quantitative findings, highlighting that about a third of parents observed that pets had become members of their family. Some adolescents treated their pets as their child, others treated them as their siblings. There was even an anthropomorphizing observation that pets also considered their humans as part of their family. These results suggest that there may be a reciprocal relationship between how an adolescent views dog owning as part of their identity and the amount of caretaking they engage in. Dogs often involve the kind of hands-on caretaking that youth can engage in (e.g., walking, physical activity), and these types of behaviors may have even increased during the pandemic [37]. If youth are highly engaged in the care of dogs, this may foster an increased sense of identity as a pet owner. Similarly, youth who may feel strongly about identifying as a dog owner and perhaps were involved in the decision around acquiring a dog, may be more motivated to engage in caretaking. Future research should explore these reciprocal relationships longitudinally to assess if there are specific, age-appropriate strategies for parents to support youth pet caretaking that can strengthen the human–animal bond.

Quantitative results also demonstrated that family relationships improved during the pandemic for pet owning families where adolescents shared a larger burden of the pet caretaking responsibilities. Of course, this relationship is not causal, and it may be that families with stronger relationships are better able to facilitate youth engagement with pets. Past studies have suggested that a telltale sign of a healthy functioning household is how much members care and interact with their animals [40]. Our qualitative interviews highlighted the nuances of these relationships, with parents revealing a heavier burden on all members of the household, especially the children, to take care of pets given the length of time everyone was spending at home during lockdown. Emerging reports during the COVID-19 pandemic have noted the added stress of multiple caregiving roles with less familial and friend support, e.g., [10], thus placing more burden on family members within households to renegotiate how to balance caretaking duties. The reciprocal relationships between pet caretaking and family communication should be explored longitudinally to assess if there are specific areas where families can be supported in maximizing communication around the human–animal bond.

Moreover, quantitative results indicated that spending time with pets was one of the top two types of strategies for coping when stressed, even more than spending time with family, technology use (e.g., watching movies, playing video games, posting on social media), exercising, or being outdoors/in nature. Particularly during the pandemic, interacting with a pet was a stress coping strategy that was still available to youth despite social restrictions. Similar to prior studies that described child pet caretaking as playing and cuddling, e.g., [38], the adolescents in our sample also were engaged in caretaking behaviors that showcased a progression in responsibilities beyond playing and affection for the child’s benefit. Some parents described their adolescents taking care of their pets in consideration of the pet’s benefit and sometimes without prompting, e.g., walking dogs, picking up toys, feeding and watering, cleaning tanks and litter boxes, entertaining pets, and cleaning up their pet’s messes (e.g., chewing on carpet, etc.).

Parents in our sample had a range of expectations about how their teens would take on the responsibilities of pet caretaking, and noted various levels of agreement and mismatch between their perceptions of the adolescent’s role in pet caretaking and the reality. Our interviews demonstrated that some parents had rather low expectations that their teens would step up and were pleasantly surprised; Other parents had higher hopes and were disappointed at their teens’ lack of motivation for pet caretaking. Adjusting expectations and flexibility to developmental changes for both the teenager and the pet may be an important feature of optimizing these relationships and warrants further exploration. Education on developmentally appropriate levels of pet caretaking and the types of responsibilities that are needed for different species of pets could be useful to families when planning the acquisition of a pet.

The level of initiative from the teens themselves point to a major developmental shift from the childhood years that focus on play, e.g., [19] to adolescents who take their pet ownership identity to another level. Adolescents’ strong preference for spending time with pets when coping with stress and their relatively high levels of social identification as a pet owner run counter to previous impressions of youth at this age as waning in interest due to societal socialization that pets are “childish” and are not as valued as human companionship, e.g., [25,26]. Similarly, parents reported overall that youth were engaged in the care of their pets and that experiencing caretaking was important in fostering the development of responsibility.

## 5. Limitations and Conclusions

Most prior work on child or adolescent interactions with their pet companions measure quality of relationship via an attachment framework, however, current measures of human–animal interaction and relationship quality do not typically take into account the fulfillment of pet caretaking responsibilities. Because parents/guardians often note that teaching responsibility is a direct reason for acquiring a pet, understanding how responsibility and caretaking links to youth perceptions of pets and family dynamics has high practical value. Although our current study does not explore pet attachment/bond distinct from responsibility and caretaking, future studies could further examine the unique and overlapping role that pet caretaking and attachment have on the quality of the adolescent-pet relationship.

In addition to not exploring pet attachment, this study has several key limitations that could be addressed in future research. While the sample was relatively diverse, participants were recruited from a specific geographic area in the United States that is comprised primarily of urban and suburban residential locations. It may be that responsibility and caretaking of animals varies in more rural communities where families are more likely to have a combination of household pets and farm animals. There may have been a self-selection bias of particular types of parents who would agree to be interviewed about pet caretaking during the pandemic. Future research should also explore both youth and parent perceptions of responsibility longitudinally to assess any age related changes in these patterns and how those changes might impact family dynamics.

We examined our research questions using a seldom used mixed-method approach in this field and we encourage other researchers to include multiple informants and methodologies to more deeply understand the phenomenon at hand. Our sample of adolescents ranged widely from tweens to teens (ages 10–17), thus subsequent work might want to further break down the nuances between early adolescent and later adolescent developmental changes in pet caretaking responsibilities and its effects on family dynamics.

## Figures and Tables

**Table 1:** Sample Description.

	Pet Owners		Dog Owners	
	N	Percent	N	Percent
Sample	567		356	
Gender				
*Male*	251	44.3	160	44.9
*Female*	312	55	196	55.1
*Other*	4	0.7	0	0
Race				
*White*	350	61.7	236	66.3
*Black*	22	3.9	10	2.8
*Hispanic*	71	12.5	37	10.4
*Asian*	16	2.8	7	2
*American Indian*	30	5.3	16	4.5
*Multiracial*	36	6.3	16	4.5
*Middle Eastern*	12	2.1	10	2.8
*Other/Unknown*	30	5.3	24	6.7
Eligibility for Free/Reduced Price School Lunch * (Yes)	225	39.7	139	39
Identity as a Pet Owner	567	44.1	356	46.3
Quality of family relationships				
*Declined*	117	20.7	69	19.4
*Stayed the Same*	289	51.1	180	50.7
*Improved*	160	28.3	106	29.9
	N	M (SD)	N	M (SD)
Age	567	13.09 (1.52)	356	13.24 (1.54)
Pet Responsibility	543	3.25 (1.24)	351	3.25 (1.18)
Spending time with pet when stressed	471	3.36 (0.92)	299	3.44 (0.82)

* Subsidized school lunch status is a proxy for lower socioeconomic status.

**Table 2:** Results of Regression Analyses for Pet and Dog Owners.

	**Identity as a Pet Owner**							
	**Pet Owner**				**Dog Owner**			
	**B**	**SE**	**Odds Ratio**	***p*-Value**	**B**	**SE**	**Odds Ratio**	***p*-Value**
Responsibility	0.12	0.07	1.13	0.099	0.19	0.1	1.21	0.043
Gender	0.13	0.18	1.14	0.46	0.32	0.22	1.37	0.156
Age	−0.28	0.06	0.76	<0.0001	−0.28	0.08	0.76	<0.0001
Free/Reduced Price Lunch	0.19	0.19	1.21	0.316	0.24	0.24	1.27	0.315
*N*	538				350			
*R2*	0.06				0.08			
	**Spending Time with Pets When Stressed**							
	**Pet Owner**				**Dog Owner**			
	**B**	**SE**	**Β**	***p*** **−Value**	**B**	**SE**	**Β**	***p*** **−Value**
Responsibility	0.1	0.03	0.14	0.004	0.08	0.04	0.12	0.044
Gender	0.07	0.08	0.04	0.415	0.12	0.1	0.07	0.232
Age	−0.04	0.03	−0.06	0.202	−0.07	0.03	−0.13	0.028
Free/Reduced Price Lunch	0.03	0.09	0.02	0.727	−0.02	0.1	−0.01	0.873
*N*	456				295			
*R2*	0.03				0.04			
	**Quality of Family Relationships**							
	**Pet Owner**				**Dog Owner**			
	**B**	**SE**	**β**	***p*** **−Value**	**B**	**SE**	**Β**	***p*** **−Value**
Responsibility	0.08	0.03	0.11	0.01	0.1	0.04	0.13	0.016
Gender	0.15	0.08	0.08	0.067	0.15	0.1	0.08	0.125
Age	−0.03	0.03	−0.04	0.342	0	0.03	0	0.956
Free/Reduced Price Lunch	−0.19	0.08	−0.1	0.023	−0.25	0.1	−0.13	0.015
*N*	537				349			
*R2*	0.03				0.04			

**Table 3:** Qualitative themes.

I. How parents socialize adolescents to take care of pets	Conversations with adolescents before getting pets
	No petcare expectations
	Lessons surrounding pet care
	COVID-19 and changing adolescent petcare expectations
II. Nature of adolescent-pet bonds	Pets as family members
	Challenges of pets in families
III. Fulfillment of pet caretaking in families with adolescents	Adolescents volunteering to take care of pets
	Blend of volunteering and assignment to take care of pets
	Adolescents assigned to take care of pets
	Parent petcare responsibilities
	Parent expectations of adolescent petcare
	Pet relationships and coping strategies
	Pet versus dog-owning adolescents

## Data Availability

De-identified datasets available upon request.
